# Microbial Resistance to Antibiotics and Biofilm Formation of Bacterial Isolates from Different Carp Species and Risk Assessment for Public Health

**DOI:** 10.3390/antibiotics12010143

**Published:** 2023-01-10

**Authors:** Nikola Puvača, Dragana Ljubojević Pelić, Miloš Pelić, Vojislava Bursić, Vincenzo Tufarelli, Luca Piemontese, Gorica Vuković

**Affiliations:** 1 Department of Engineering Management in Biotechnology, Faculty of Economics and Engineering Management in Novi Sad, University Business Academy in Novi Sad, Cvećarska 2, 21000 Novi Sad, Serbia; 2 Scientific Veterinary Institute Novi Sad, Rumenački Put 20, 21000 Novi Sad, Serbia; 3 Faculty of Agriculture, University of Novi Sad, Trg Dositeja Obradovića 8, 21000 Novi Sad, Serbia; 4 Department of Precision and Regenerative Medicine and Jonian Area (DiMePRe-J), Section of Veterinary Science and Animal Production, University of Bari ‘Aldo Moro’, 70010 Bari, Italy; 5 Department of Pharmacy—Pharmaceutical Sciences, University of Bari Aldo Moro, 70125 Bari, Italy; 6 Faculty of Agriculture, University of Belgrade, 11080 Belgrade, Serbia

**Keywords:** antimicrobial resistance, risk assessment, antibiotics, fish, carp, biofilm, food

## Abstract

The aim of this research was to investigate the effects of biofilm on antibiotic resistance of the bacterial isolates present in fish meat and to assess the risk of antibiotic residues for public health. Common carp, silver carp and grass carp fishes were purchased from retail stores for an in vitro biofilm investigation and a drug-resistant pattern determination. In all samples, up to 10^4^ CFU/g of bacteria, such as *Escherichia coli, Aeromonas hydrophila, Shewanella putrefaciens, Vibrio* spp. and *Staphylococcus* spp., were observed. Isolates from the samples and their biofilms were subjected to an antibiogram assay using antibiotics such as amoxicillin, ampicillin, cefotaxime, ciprofloxacin, chloramphenicol, gentamicin, streptomycin, tetracycline and trimethoprim. Obtained results showed that some of the isolates were sensitive to antibiotics and some were resistant. Results of LC-MS/MS analysis showed that antibiotics residues were present in fish samples in the range between 4.9 and 199.4 µg/kg, with a total sum of 417.1 µg/kg. Estimated daily intake (EDI) was established to be 0.274 μg/kg of body weight/day for men and 0.332 μg/kg of body weight/day for women, with an acceptable daily intake (ADI) of 8.5 and 7.0 µg/kg of body weight/day for men and women, respectively. The results of the present study, therefore, highlight the safe consumption of fresh fish.

## 1. Introduction

Foodborne pathogens are one of the main sources of infectious diseases throughout the world [1,2]. Their ability to adapt and survive in food habitats is largely due to the biofilms formed by indigenous microbiota [3,4,5] and several species of bacteria form biofilms that unconditionally cause foodborne diseases [6]. Biofilm formation mechanisms [7] are undoubtedly becoming an important research area [8,9,10] and indeed, contemporary human and veterinary medical practices, as well as food processing protocols, comprise several intervention strategies to hinder biofilm formation [11,12]. Moreover, foodborne pathogens that produce biofilms can carry genes that confer antibiotic resistance, making them an even greater threat to food safety [13].

A disease outbreak frequently occurs in intensive fish farming, particularly in the spring when fingerlings are hatched [14]. Recently the use of antibiotics in aquaculture has moved beyond the mere purpose of preventing and treating fish infections. In fact, some of these bioactive substances, in sub-inhibitory concentrations, are now used in various countries as feed additives with the aim of promoting growth [15]. However, this possibility is questioned considering that the presence of trace antibiotic residues in fish meat is a possible danger to human health, both directly, due to possible multiple toxicities and indirectly, because it causes the emergence of antibiotic resistance. A common carp (*Cyprinus carpio*) is the most important fish in Serbian and overall European aquaculture. Other species such as silver carp (*Hypophthalmichthys molitrix*) and grass carp (*Ctenopharyngodon idella*) are usually grown in the polyculture with common carp [16]. Various food [17,18] and water [19,20] tests have identified a significant number of drug-resistant strains, along with multidrug-resistant (MDR) strains, indicating a potential ecological spread [21].

Antibiotic resistance is therefore a worldwide threat [22], especially in developing countries with a high density of population below the poverty line, shaded primarily by pathogenic microbes [23] where responsible and prudent use of antibiotics is left out. Researchers have shown that when monospecies or different species form biofilms and communicate with one another via quorum sensing, they can transfer resistant genomic materials [24,25,26,27,28]. By forming biofilm in a specific food, foodborne pathogens can increase antibiotic resistance [29,30]. Many studies have concentrated on isolated species or pure cultures, but biofilms can consist of many different bacterial species as well as fungi [31], algae [32] and protozoa [33] in natural environments. As a result of their greater resistance to disinfectants and sanitizers, mixed biofilms have recently become more important in food microbiology and food protection [4,34]. Moreover, multispecies biofilms may offer expanded opportunities for interaction in natural habitats, including horizontal gene transfer and cometabolism, which may increase drug resistance [28,35,36].

There are two major adverse effects on human health caused by the extensive use of antibiotics in animals, such as bacterial resistance and toxicological effects [37,38,39,40,41,42]. The issue of bacterial resistance is forcing modern medicine into the post-antibiotic era. It is also possible that the resistance genes in animal microbiomes on human microbiota can be horizontally transferred [43,44]. Toxicological effects have been extended to immune and metabolic diseases due to the emergence of a link between the human microbiome and physiological function [45].

In general, humans are exposed to antibiotics primarily through their clinical use and food and water residues [19,46,47]. It is better to explore the toxicological effects of antibiotics by examining how they are used in various settings. According to some studies, adults and children were exposed to multiple human or veterinary antibiotics [48], where drinking water may not have been a major source of exposure. Despite this, there are no comprehensive data available about antibiotic residues in food and their impact on human health. Most studies have focused on antibiotic resistance of bacteria isolated from raw samples of various foods, including vegetables [8,18,49].

Taking into consideration all of the above, this study investigated the initial bacterial load from the fish samples and evaluated the ability of the isolates to form biofilms and assessed the risk for human daily fish consumption with residues of antibiotics.

## 2. Results and Discussion

It has been shown that contaminated food can spread resistant bacteria to consumers. Researchers have analyzed bulk water [50], fish [51], vegetables [52], dairy products [53], eggs [54] and meat [55] samples to sort out drug-resistant bacteria and their distribution in the environment. Today, scientists are exploring the formation of mixed-species biofilms in the environment as a result of close bacterial interactions.

### 2.1. Microbiological Quality of the Tested Fresh Fish Samples

Examined bacteria in a range of 10^2^–10^4^ CFU/g (Table 1) were found in all tested samples of common carp, silver carp and grass carp. The present study estimated 6 isolates (*E*. *coli*, *A*. *hydrophila*, *Salmonella* spp., *Shewanella putrefaciens*, *Vibrio* spp. and *Staphylococcus* spp.) from 3 categories of 15 carp fish samples. Among the six isolates, *E*. *coli*, *A*. *hydrophila*, *Vibrio* spp. and *Staphylococcus* spp. were found in every category of samples within the range of 10^2^–10^4^ CFU/g while *Shewanella putrefaciens* was found up to 10^2^ CFU/g in only silver carp (Table 1). *Salmonella* spp. was not isolated from examined samples.

As quantified in early findings, freshwater fish, as well as sea fish, can be contaminated with several pathogenic and commensal bacteria (*Salmonella* spp., *E. coli, Proteus* spp., *Staphylococcus* spp., *Klebsiella* spp., *Shigella* spp., *Pseudomonas* spp., *Listeria* spp. and *Vibrio* spp.) and fungi. In the case of examined carp species biofilm, only *E. coli, A. hydrophila* and *Staphylococcus* spp. were identified, whereas *Shewanella putrefaciens, Vibrio* spp. and *Salmonella* spp. were not found in the biofilm. Several reasons are responsible for the missing growth of some species after biofilm formation. Firstly, during 7 days of incubation, the supplied nutrients gradually declined and the condition enforced some bacteria to go forward to the death phase. Secondly, in the environment, one species always tries to inhibit the growth of another species by producing some toxins or antibiotics for their nutrients and survival. From the clinical point of view, the presence of such pathogenic bacteria has an enormous possibility of prolonging the enteric diseases of consumers. Similar results were also reported by Cai and Arias [10] from the fish samples.

### 2.2. Antibiotic Resistance of the Isolates from Different Carp Biofilms

From the results shown in Table 2, Table 3 and Table 4, it can be noticed that the bacterial isolates, which already have been sensitive to antibiotics used in the test, exhibited resistance against that antibiotic in every case when they were isolated from biofilms. Obtained results have been confirmed by the antibiogram test, respectively. All the isolates from both bulk and biofilms showed resistance against ampicillin (AM) and tetracycline (TE). When bacteria were isolated from the biofilm, multidrug resistance frequency was highly accelerated.

If tests had been examined more carefully, some of the isolates would have been vulnerable to other antibiotics. The isolates from common carp samples, *E. coli*. and *Staphylococcus* spp. exhibited sensitivity against streptomycin (STR) and trimethoprim (TMP) and resistance against AM and TE but gained resistance against that antibiotics when they were grown in biofilm (Table 2).

In the case of silver carp samples, biofilm isolates of *E. coli* gained resistance against amoxicillin (AMC), AM and TE and showed intermediate results for STR in bulk samples but showed resistance against STR in biofilm (Table 3). Isolates of *Vibrio* spp. from bulk samples were resistant against AMC, STR and TE and *Staphylococcus* spp. were resistant against AMC, AM, STR and TE, while the rest of the isolates showed sensitivity. Isolates of *E. coli*, *A. hydrophila*. and *Staphylococcus* spp. from the biofilm acquired their resistance against AMC, AM, STR and TE, respectively.

In the case of grass carp samples, *E. coli* from the bulk samples were found to be resistant to all the antibiotics, such as AMC, AM and TE, while *Staphylococcus* spp. exhibited resistance against AM and TE. Furthermore, *A. hydrophila*., *Shewanella putrefaciens, Vibrio* spp. and *Staphylococcus* spp. were found to be sensitive to all the antibiotics. The same species (*Vibrio* spp. and *Staphylococcus* spp.) from the biofilm of the same samples turned into resistance against all the antibiotics (Table 4).

In most cases, the sensitive pathogen became resistant to most of the antibiotics tested when they were isolated from the mixed-species biofilm. The results in this study indicate that multidrug resistance biofilms may engage effectively in the regular distribution of antibiotic resistance genes in the natural world, resulting in an increased incidence of multidrug resistance cases and thus increasing the risk to public health.

The maximum amount of antibiotic residue allowed in foods of animal origin is between 50 and 500 µg/kg, according to Commission Regulation (EU) No 37/2010 [56] of 22 December 2009 on pharmacologically active substances and their classification regarding maximum residual levels (MRLs). Our findings showed differences in the residual antibiotics in fish meat samples (Table 5). These fishes can be safely used in human nutrition, keeping in mind the limits of each separate antibiotic according to Commission Regulation (EU) No 37/2010 [56], but from the consumer point of view, these fishes are “not antibiotic-free”.

Fish is one of the very popular foods for a healthy lifestyle in Europe [57,58,59,60]. An increase in antibiotic resistance genes may be caused even by low-level doses of antibiotics consumed by fish over a long time [61]. Thus, the health effects of antibiotic consumption in the diet should be evaluated. To ensure that residue levels are not unacceptable, exporting countries must provide guarantees that monitoring is conducted, while antibiotic residue limits have been established in the EU for animal products [56]. For both freshwater and sea species, it is important to develop and regulate appropriate withdrawal periods for these substances. It was possible to harvest and commercialize fish after the withdrawal period was over. In our research, from a risk assessment perspective, fish samples were collected randomly from retail stores.

**Figure 1:**
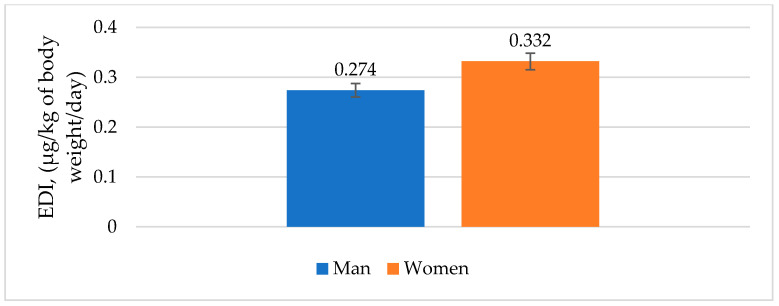
Estimated daily intake (EDI) through freshwater fish consumption by men and women in Europe.

**Figure 2:**
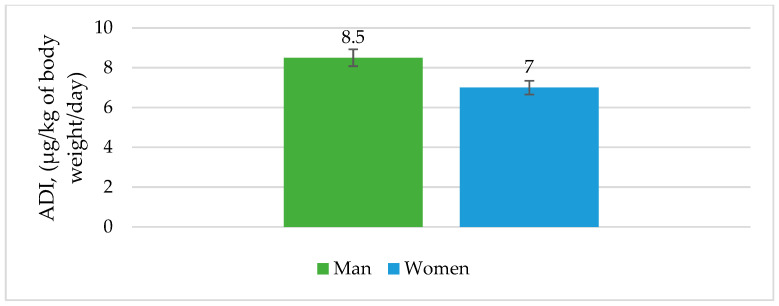
Acceptable daily intake (ADI) through freshwater fish consumption by men and women in Europe.

As shown in Figure 1 and Figure 2, the calculated estimated daily intake (EDI) and acceptable daily intake (ADI) were presented separately for men and women.

The calculated EDI for freshwater fish in our research was 0.274 μg/kg of body weight/day for men and 0.332 μg/kg of body weight/day for women. The ADI value of antibiotics residues in fish meat was calculated to be 8.5 and 7.0 μg/kg of body weight/day for men and women, respectively. If fish were a major component of the diet, the contribution of investigated fish to dietary intake of total antibiotic residues would not seem to pose a risk to public health, according to the calculated EDI results. Despite limited toxicological data and approved standards, it is still difficult to estimate antibiotics’ potential risks to public health.

In our research, the majority of antibiotic residues were under MRLs, but according to Wang et al. [62], people who consume meat, milk and aquatic products daily might experience a long-term mixed exposure mode to multiple antibiotics of low-dose less than 1 μg/kg/day. In earlier studies by the same authors [63], adverse effects of level antibiotics in urines were associated with children’s obesity, suggesting multiple potential health risks from antibiotic residues in used food products.

## 3. Materials and Methods

### 3.1. Design of Study and Fish Sampling

A total of 15 samples of 3 species of fish samples (*n* = 5 per species) such as common carp, silver carp and grass carp were randomly collected from the different local fish markets and supermarkets in Novi Sad during November 2019 and transported immediately to the laboratory and stored till further analyses. For the isolation and enumeration of bacteria, 10 g of each sample was homogenized in 90 mL normal saline and diluted up to 10^−5,^ and aliquots were seeded onto tryptone soya agar plates by spread plate technique in duplicate. The color, opacity, shape and structure of colonies were used to identify bacterium types. A pure culture was also obtained by streaking five colonies onto tryptone soya agar plates.

### 3.2. Isolation and Identification of Bacteria

For the final identification of bacteria, API 20E tests were used. A variety of tests had been conducted before this, including Gram stain, morphology, motility, catalase, oxidase, lipase, indole, H_2_S production, gelatinase and nitrate reduction.

### 3.3. Antibiotic Susceptibility

The Kirby Bauer technique (the standard agar-disc-diffusion method) was used to determine the sensitivity to antibiotics of pathogenic bacteria in Mueller–Hinton agar (Difco, Detroit, MI, USA) collected directly from carp samples. A wide range of antibiotics was used, many of which were widely available, such as amoxicillin (AMC, 20 µg), ampicillin (AM, 10 µg), cefotaxime (CTX, 30 µg), ciprofloxacin (CIP, 5 µg), chloramphenicol (CHL, 30 µg), gentamicin (CN, 10 µg), streptomycin (STR, 10 µg), tetracycline (TE, 30 µg) and trimethoprim (TMP, 5 µg). Incubation of inverted plates was set for 24 h at 37 °C, upon which the plates were examined, following the measure of and the zone of inhibition (mm) [64].

### 3.4. In Vitro Biofilm Formation and Recovery

For the in vitro biofilm formation, 10 g of each fish muscle sample was added into 90 mL of nutrient broth and placed for shaking into the wise bath at 37 °C at 120 rpm for 5 to 7 days. Bacteria living free in each sample were permitted to expand in the broth medium before nutrients that reflected the selective pressure of the development of biofilms were decreased. Biofilm formation was demonstrated by the thin, opaque coating suspended on the surface of the medium anchor flask [65].

The thin layers of free-living bacteria were further revived through the selective stick plate methods. Isolation and identification of bacteria were performed in the same way as previously described.

### 3.5. Antibiotic Susceptibility Pattern of the Isolates after Biofilm Formation

The antibiotic susceptibility pattern of the bacteria from the biofilm matrix was introduced on Mueller–Hinton agar (Difco, Detroit, MI, USA) against the same set of antibiotics to examine the bacterial resistance. Plates were again incubated at 37 °C for 24 h, as previously described and properly examined, followed by the zone of inhibition measurement.

### 3.6. Analysis of Antibiotics

Antibiotics in carp meat samples were determined by LC-MS/MS previously described in detail by Puvača et al. [18]. Sampled fish fillets were minced and then stored in polypropylene bottles at −18 °C until analyzed. The aliquot of 2.0 g of each sample was weighed into a 50 mL polypropylene tube, with 8 mL of acetonitrile added and the sample was homogenized. The sample was centrifuged and the supernatant was decanted into a 15 mL polypropylene tube. The volume of the extract was brought up to 10 mL with distilled water and 0.3 g of dispersive C18 was added to it. The supernatant was shaken for 1 min and then centrifuged for 5 min. Afterward, a 1 mL aliquot was diluted with 1 mL of distilled water and the extract was passed through a PTFE membrane filter. The final extract corresponded to a 0.1 g/mL sample equivalent. LC-MS/MS was performed with a spiking level of 0.1 mg/kg and the test data were evaluated based on recovery percentage (%) and relative standard deviation (SD).

### 3.7. Risk Assessment for Fish Consumption

The daily estimation of the exposure to antibiotics from fish meat for adults is based on their average daily consumption. To evaluate chronic effects on public health, the maximum residue limit (MRL) set by the European Commission is used [56]. Food and Agriculture Organization and World Health Organization (FAO/WHO) recommended acceptable daily intakes (ADIs) as percentages of estimated daily intakes (EDIs) daily, while ADIs were calculated based on a mice model for carcinogenicity: NOAEL = 10 mg/kg of body weight/day. To calculate the EDIs of the sum of antibiotic residues, the following formula was used:

EDI = (C × K)/BW

where

EDI—estimated daily intake (μg/kg of body weight/day);

C—the average concentration of antibiotics in fish meat (μg/kg);

K—average consumption rate (kg of fish/day);

BW—average human body weight (kg).

The data on average daily fish meat consumption by adults were based on European Commission for major world economies in the year 2019 [66]. The daily consumption of fish meat in the year 2019 was set as the normal distribution with a mean of 0.056 kg. The body weight of European adults aged 20 years and above was set as the normal distribution with a mean of 85.08 kg (±1.83) for men and 70.15 kg (±2.60) for a woman, according to the World Data Info [67].

## 4. Conclusions

As a result of our research, a wide variety of microorganisms were identified, including pathogenic ones. In all samples of fish tested, pathogens were found, indicating health risks when consumed. Multidrug-resistant bacteria may be spread by both pathogens and commensal microorganisms when mixed-species biofilms exist. It was found that mixed species biofilms formed in vitro from the same samples were antibiotic-resistant.

Results of LC-MS/MS analysis have shown that antibiotics residues were present in fish samples in the range between 4.9 and 199.4 µg/kg, with a total sum of 417.1 µg/kg. Estimated daily intake (EDI) was established to be 0.274 μg/kg of body weight/day for men and 0.332 μg/kg of body weight/day for women, with an acceptable daily intake (ADI) of 8.5 and 7.0 µg/kg of body weight/day for men and women, respectively.

The results of the present study, therefore, highlight the safe consumption of fresh fish.

## Figures and Tables

**Table 1:** Isolation and quantification of pathogenic bacteria from common carp, silver carp and grass carp samples (*n* = 5).

Species	Microbial Load (CFU/g)					
	*E. coli*	*A. hydrophila*	*Salmonella* spp.	*Shewanella putrefaciens*	*Vibrio* spp.	*Staphylococcus* spp.
Common carp	3.3 × 10^3^	8.8 × 10^3^	nd	nd	1.3 × 10^3^	1.0 × 10^2^
Silver carp	6.9 × 10^4^	7.8 × 10^4^	nd	1.6 × 10^2^	3.6 × 10^2^	2.2 × 10^3^
Grass carp	4.4 × 10^4^	6.6 × 10^4^	nd	nd	1.8 × 10^2^	1.2 × 10^2^

nd—not detected. All analyses were performed in triplicate.

**Table 2:** Antibiotics susceptibility pattern of the isolates from common carp.

Isolates		Antibiotics								
		AMC	AM	CTX	CIP	CHL	CN	STR	TE	TMP
**Bulk sample**	*E. coli*	I	R	S	S	S	S	S	R	S
	*A. hydrophila*	I	R	S	S	S	S	S	R	S
	*Vibrio* spp.	I	R	S	S	S	S	S	R	S
	*Staphylococcus* spp.	S	R	S	S	S	S	S	R	S
**Biofilm**	*E. coli*	R	R	S	S	S	S	R	R	R
	*Staphylococcus* spp.	R	R	S	S	S	S	I	R	I

R—Resistant; I—Intermediate; S—Sensitive; AMC– Amoxicillin (20 µg); AM—Ampicillin (10 µg); CTX—Cefotaxime (30 µg); CIP—Ciprofloxacin (5 µg); CHL—Chloramphenicol (30 µg); CN- Gentamicin (10 µg); STR—Streptomycin (10 µg); TE—Tetracycline (30 µg); TMP—Trimethoprim (5 µg).

**Table 3:** Antibiotics susceptibility pattern of the isolates from silver carp.

Isolates		Antibiotics								
		AMC	AM	CTX	CIP	CHL	CN	STR	TE	TMP
**Bulk sample**	*E. coli*	R	R	S	S	S	S	I	R	S
	*A. hydrophila*	S	S	S	S	S	S	S	R	S
	*Shewanella putrefaciens*	S	S	S	S	S	S	S	S	S
	*Vibrio* spp.	R	I	S	S	S	S	R	R	S
	*Staphylococcus* spp.	R	R	S	S	S	S	R	R	S
**Biofilm**	*E. coli*	R	R	S	S	I	S	R	R	I
	*A. hydrophila*	R	R	S	S	S	S	R	R	I
	*Staphylococcus* spp.	R	R	S	S	S	S	R	R	I

R—Resistant; I—Intermediate; S—Sensitive; AMC– Amoxicillin (20 µg); AM—Ampicillin (10 µg); CTX—Cefotaxime (30 µg); CIP—Ciprofloxacin (5 µg); CHL—Chloramphenicol (30 µg); CN- Gentamicin (10 µg); STR—Streptomycin (10 µg); TE—Tetracycline (30 µg); TMP—Trimethoprim (5 µg).

**Table 4:** Antibiotics susceptibility pattern of the isolates from grass carp.

Isolates		Antibiotics								
		AMC	AM	CTX	CIP	CHL	CN	STR	TE	TMP
**Bulk sample**	*E. coli*	R	R	S	S	S	S	S	R	S
	*A. hydrophila*	S	S	S	S	S	S	S	S	S
	*Shewanella putrefaciens*	S	S	S	S	S	S	I	S	S
	*Vibrio* spp.	S	S	S	S	S	S	I	S	S
	*Staphylococcus* spp.	S	R	S	S	S	S	I	R	S
**Biofilm**	*E. coli*	R	R	S	S	I	S	R	R	I
	*A. hydrophila*	R	R	S	S	S	S	R	R	I

R—Resistant; I—Intermediate; S—Sensitive; AMC– Amoxicillin (20 µg); AM—Ampicillin (10 µg); CTX—Cefotaxime (30 µg); CIP—Ciprofloxacin (5 µg); CHL—Chloramphenicol (30 µg); CN- Gentamicin (10 µg); STR—Streptomycin (10 µg); TE—Tetracycline (30 µg); TMP—Trimethoprim (5 µg).

**Table 5:** Average contents of antibiotics in fish samples (*n* = 15).

	Antibiotics									Sum of Antibiotics
	AMC	AM	CTX	CIP	CHL	CN	STR	TE	TMP	
Concentration, µg/kg	11.4	4.9	-	52.3	-	31.1	199.4	92.4	25.6	417.1
SD	0.02	0.00	-	0.28	-	0.43	0.95	0.18	0.02	
Recovery, %	90.12	89.31	-	90.22	-	78.61	81.33	95.12	98.38	

AMC– Amoxicillin (20 µg); AM—Ampicillin (10 µg); CTX—Cefotaxime (30 µg); CIP—Ciprofloxacin (5 µg); CHL—Chloramphenicol (30 µg); CN- Gentamicin (10 µg); STR—Streptomycin (10 µg); TE—Tetracycline (30 µg); TMP—Trimethoprim (5 µg); SD—Standard deviation.

## Data Availability

Data are provided in the article.
